# Factors associated with self-care in older adults with heart failure presenting with composite and domain-specific intrinsic capacity impairment

**DOI:** 10.3389/fcvm.2026.1996296

**Published:** 2026-10-29

**Authors:** Yanhua Xu, Wenting Xu, Xiaojing Li, Ronggang Peng, Qing Zhao

**Affiliations:** 1 Department of Cardiology, Affiliated Wuxi People's Hospital of Nanjing Medical University, Wuxi People's Hospital, Wuxi Medical Center, Nanjing Medical University, Wuxi, China; 2 Department of Cardiac Care Unit, Affiliated Wuxi People's Hospital of Nanjing Medical University, Wuxi People's Hospital, Wuxi Medical Center, Nanjing Medical University, Wuxi, China; 3 Department of Cardiology, Affiliated Hospital of Jiangnan University, Wuxi, China

**Keywords:** heart failure, intrinsic capacity, older adults, self-care, observational study

## Abstract

**Objective:**

This study aimed to characterize intrinsic capacity (IC) impairment in older adults with heart failure and to investigate its association with self-care across composite and domain-specific domains.

**Methods:**

This cross-sectional study recruited 215 patients with heart failure from two tertiary hospitals in Wuxi, China. IC was assessed across five domains in line with WHO guidelines: cognition, locomotion, vitality, sensory, and psychological. Self-care was evaluated using the Self-Care Heart Failure Index version 6.2. Data were analyzed using descriptive statistics, Pearson correlation coefficients, multiple linear regression, and exploratory interaction analyses.

**Results:**

Our study found that 99.5% of participants exhibited impairment in at least one IC domain, with the most common co-occurring impairment in cognition, vitality, and locomotor domains. Older age was significantly associated with poorer self-care maintenance (B = −0.37, 95% CI [−0.67, −0.07], *P* = 0.014), self-care management (B = −0.64, 95% CI [−1.02, −0.26], *P* = 0.001), and self-care confidence (B = −0.38, 95% CI [−0.77, −0.0004], *P* = 0.049). Higher educational level was significantly associated with better self-care maintenance (B = 9.80, 95% CI [3.87, 15.73], *P* = 0.001). A higher composite IC score was significantly associated with poorer maintenance (B = −2.81, 95% CI [−4.68, −0.95], *P* = 0.003) and lower confidence (B = −3.53, 95% CI [−5.95, −1.11], *P* = 0.004), but not with management. Domain-specific analyses showed that cognitive, psychological, and sensory impairments were associated with poorer self-care maintenance, whereas cognitive, locomotor, and psychological impairments were associated with lower self-care confidence. No IC domain was significantly associated with self-care management. Exploratory interaction analyses identified several potential moderating effects involving age, New York Heart Association class and education for associations between IC impairments and self-care.

**Conclusion:**

Healthcare professionals should routinely assess IC in older adults with heart failure. Addressing multimodal intrinsic capacity impairment, particularly among the oldest patients and those with lower educational attainment, may help improve self-care.

## 1 Introduction

Heart failure is a prevalent chronic condition among older adults and imposes substantial symptom burden, physical debilitation, and reduced quality of life (1). The prevalence of heart failure increases with population aging. Adults aged 60 years and older have approximately a 20-fold higher risk of developing heart failure than that of younger individuals (2). Heart failure is frequently accompanied by multimorbidity, polypharmacy, and functional limitations. These challenges complicate disease management and contribute to poor clinical outcomes (3).

Self-care is central to effective heart failure management. It encompasses a set of behaviors such as monitoring symptoms and body weight, managing medication effects, adhering to dietary and fluid restrictions (self-care maintenance), seeking medical assistance for acute symptoms (self-care management), and confidence in managing worsening symptoms (self-care confidence) (4). Effective self-care is associated with better quality of life and could reduce up to 30% of hospital admissions and more than 50% of readmissions in patients with heart failure (5). Despite these benefits, heart failure self-care remains inadequate due to multiple barriers, such as older age, lower education, multimorbidity, disease severity, cognitive impairment, and depressive symptoms (6, 7).

Intrinsic capacity (IC), introduced by the World Health Organization, describes an individual’s composite physical and mental capacities. This multidimensional healthy-ageing framework may explain heterogeneity in self-care among older adults with heart failure. IC is commonly operationalized across locomotion, cognition, psychological well-being, vitality, and sensory domains (8). This multidomain perspective is particularly relevant in heart failure, a condition characterized by complex interactions across cardiovascular, musculoskeletal, and neurocognitive systems (9). Cardiac dysfunction, hemodynamic instability, and reduced exercise tolerance can directly compromise locomotion and vitality (10). Heart failure-related cerebral hypoperfusion, inflammation, and coexisting vascular disease may impair cognition (11). The psychological burden of living with a chronic illness and social isolation can manifest as depression, while age-related sensory impairment further reduces the process health-related information. Together, these changes erode IC, reducing physiological resilience and contributing to higher mortality, more healthcare utilization, and increased risk of incident cardiovascular events (12, 13).

Impairments across IC likely constrain self-care in heart failure. The situation-specific theory of heart failure self-care indicated that cognitive impairment, depression, reduced physical function, and poor nutritional status closely related to self-care (6). These factors closely correspond to IC domains, suggesting a potentially critical role of IC in understanding the correlates of self-care in older adults with heart failure. Indeed, effective self-care likely relies on preserved IC. Specifically, cognitive function supports learning and executing complex treatment plans, physical capacity enables the actions required for daily self-care, psychological well-being influences motivation and adherence, vitality provides the energy for sustained participation, and sensory function supports independent functioning. These domains do not operate in isolation but collectively shape patients’ ability to initiate, adapt, and maintain self-care. Consistent with this perspective, composite IC impairment has been linked to poor self-care management among older adults (14).

Despite these converging lines of evidence, research linking IC impairment to self-care in older adults with heart failure remains limited. Findings from general older populations may not be directly generalizable to individuals with heart failure, given their distinct pathophysiology, symptom burden, and treatment complexity. Most heart failure studies focused on single IC-related factors, such as physical performance, cognitive impairment, or depression, rather than adopting a comprehensive IC framework. In addition, although sociodemographic factors (e.g., age, education, monthly income) and clinical variables (e.g., comorbidities, disease severity) are consistently identified as predictors of self-care (6), they are rarely examined within the context of IC. Prior work also rarely distinguishes between composite IC impairment (reflecting an overall impairment across multiple domains) and domain-specific IC impairment, which may have distinct and interactive influences on self-care. To date, the association between IC, both as a composite score and domain-specific impairment, and self-care among older adults with heart failure remains insufficiently explored.

To address these gaps, this study aims to investigate the factors associated with self-care among older adults with heart failure presenting with both composite and domain-specific IC impairments. Specifically, we intend to: (1) characterize IC impairment and self-care in this population; (2) examine the relationship between composite and domain-specific IC impairment and heart failure self-care; and (3) explore whether domain-specific IC impairments interact with patient characteristics on self-care. Clarifying these associations may inform personalized and preventive care strategies for older adults living with heart failure.

## 2 Materials and methods

### 2.1 Study design

This was an observational cross-sectional study reported in accordance with the Strengthening the Reporting of Observational Studies in Epidemiology (STROBE) guideline for cross-sectional studies (15).

### 2.2 Participants

The convenience sampling was adopted to recruit hospitalized patients with heart failure from the Departments of Cardiology of two tertiary hospitals in Wuxi, China, between June 2023 and May 2024. Participants were eligible if they were: (1) aged 60 years or older; (2) have physician-confirmed diagnosis of heart failure; (3) have New York Heart Association (NYHA) Class II to IV, as these participants were symptomatic with activity limitation, within which IC domains may be more likely to be driven by heart failure-related pathophysiological processes (e.g., neurohormonal activation, systemic inflammation, or reduced cardiac output); and (4) were able to comprehend and communicate in Chinese, to ensure the validity of the interview-based assessment and self-report measures. Participants with severe liver or kidney dysfunction, advanced tumors, or those participating in interventional studies were excluded, as these conditions may substantially affect IC, functional status, and self-care, thereby confounding the interpretation of the observed associations. This study was approved by the Ethics Committee of Wuxi People's Hospital (Approval No. 2023-KY23114), and written informed consent was obtained from participants before data collection.

Sample size was estimated *a priori* using G*Power 3.1 for multiple linear regression. Based on Cohen's convention, a medium effect size was assumed (f^2^ = 0.15) (16), with a two-sided significance level of 0.05, statistical power of 0.90, and 18 candidate predictors. The minimum required sample size was 183 participants. To account for approximately 10% incomplete or unusable data, the target sample size was increased to 204. Ultimately, 215 participants were enrolled, exceeding the required sample size and providing sufficient power for the planned analyses.

### 2.3 Measures

#### 2.3.1 Socio-demographic and clinical characteristics

Socio-demographic and clinical information was collected through patient interviews and review of medical records. Collected data included age, body mass index (BMI), gender, marital status, educational level, monthly income, living arrangements, medical expense coverage, NYHA functional classification, current smoking status, current drinking status, and history of chronic diseases (hypertension, coronary heart disease, diabetes, stroke, chronic obstructive pulmonary disease, and tumor).

#### 2.3.2 IC impairment assessment

In line with the WHO ICOPE framework, which comprises a two-step approach (Step 1 screening and Step 2 in-depth assessment), IC was evaluated across five domains: cognition, locomotor, vitality, psychological well-being, and sensory capacity (17). Each domain was operationalized using the in-depth assessment instruments recommended in the WHO ICOPE Step 2 guidance. Each IC domain was coded based on impairment status, with a score of 1 indicating an impaired domain and 0 indicating no impairment. A composite IC score was derived by summing the number of impaired domains, with the total score ranging from 0 (no domains impaired) to 5 (all domains impaired). Higher composite scores indicate greater impairment and worse IC. The following instruments and cut-off values are used to define each domain of IC impairment in this study, which are consistent with the WHO ICOPE Step 2 detailed assessment recommendations (17).

Cognitive domain was measured using the Montreal Cognitive Assessment (MoCA), which was originally developed by Nasreddine (18), and later translated and adapted into the Beijing version (MoCA-BJ) by Yu et al. (19). This scale comprises visuospatial/executive function, naming, memory, attention, language, abstraction, delayed recall, and orientation. The total MoCA score ranges from 0 to 30, with higher scores indicating better cognitive performance. The internal consistency reliability (Cronbach's *α*) of the MoCA-BJ is 0.88, and its criterion-related validity with the Mini-Mental State Examination is 0.83 (19). Cognitive impairment was defined as a MoCA score <26, a cutoff widely used for detecting cognitive impairment in older populations (19).

Locomotor domain was evaluated using the Short Physical Performance Battery (SPPB), originally developed by Guralnik et al. (20), which assesses standing balance, gait speed, and chair-stand performance on a 0–12 scale. Higher scores indicate better locomotor function. SPPB has been demonstrated to have good validity, with intraclass correlation coefficients ranging from 0.82 to 0.92 (21). Based on the SPPB manual, a total score ≤ 9 was used to define locomotor impairment (20).

Vitality domain of IC was assessed using the Mini Nutritional Assessment-Short Form (MNA-SF), which is recommended by the European Society for Clinical Nutrition and Metabolism for use in older adults (22). MNA-SF was originally developed by Rubenstein (23), comprising six items that assess food intake, weight loss, mobility, presence of acute disease or psychological stress, neuropsychological problems, and body mass index. The total score ranges from 0 to 14, and scores ≤11 were considered indicative of impaired vitality (24).

Psychological domain was assessed using the 15-item Geriatric Depression Scale (GDS-15), which was originally developed by Yesavage (25), and later adapted for the Chinese context based on population characteristics (26). The total score of this scale ranges from 0 to 15, with higher scores indicating worse depressive symptoms. The internal consistency reliability was 0.89, and concurrent validity with CES-D was 0.96 for the Chinese adaptation (26). Impairment in psychological well-being was defined as GDS-15 score ≥ 5 (25).

Sensory domain was assessed based on self-report. Participants were asked whether they had experienced any difficulty seeing or hearing that affected daily functioning. A positive response in vision or hearing was classified as an impairment in sensory capacity.

#### 2.3.3 Self-care

Self-care was assessed using the 22-item Self-Care Heart Failure Index version 6.2 (SCHFI v6.2), which was originally developed by Riegel (27), and later translated into the Chinese version by Kang (28). This scale measures self-care across three subscales: self-care maintenance (10 items), self-care management (6 items), and self-care confidence (6 items). Scores of each subscale were standardized to a scale of 0 to 100, with a score of 70 or higher indicating adequate self-care. Standardized subscale scores were calculated as: self-care maintenance, (sum of items-10) × 3.333; self-care management, (sum of items-4) × 5; and self-care confidence, (sum of items-6) × 5.56 (27). The Chinese version of the SCHFI v6.2 has demonstrated acceptable internal consistency, with Cronbach's *α* values of 0.66 for self-care maintenance, 0.74 for self-care management, and 0.87 for self-care confidence. The criterion-related validity of the Chinese version of the SCHFI v6.2 with Partner in Health scale ranged from −0.35 to −0.60 (28).

### 2.4 Data collection procedure

Eligible participants were approached during hospitalization and informed about the study objectives. After written informed consent was obtained, data were collected by trained researchers within 24 h before discharge. For participants with limited literacy or impaired vision, the researcher read each questionnaire item aloud and assisted in recording their responses. All completed questionnaires were checked for completeness and consistency before being entered into the database for analysis.

### 2.5 Data analysis

Statistical analyses were performed using IBM SPSS Statistics 20.0. Normality of continuous variables was assessed using skewness and kurtosis coefficients, with the absolute values of skewness <2 and kurtosis <7 indicating approximate normality. Age (skewness = 0.213, kurtosis = 0.394), BMI (skewness = 0.026, kurtosis = 0.179), composite IC (skewness = 0.250, kurtosis = 0.572), and self-care maintenance, management, and confidence (skewness range: 0.152 to 0.646; kurtosis range: 0.002 to 0.599) all met these criteria and were therefore treated as normally distributed.

Continuous variables are presented as mean ± standard deviation (SD), and categorical variables as frequency (N) and percentage (%). Co-occurring IC impairments were defined as impairment in a specific subset of domains with no impairment in any remaining domain and were visualized using an UpSet plot in R version 4.5.3. Differences in self-care across various socio-demographic and clinical characteristics, and each domain in IC, were assessed using independent-samples t-tests and one-way ANOVA. Pearson's correlation analysis was conducted to explore the linear relationships between age, BMI, composite IC score, and self-care.

Multiple linear regression analyses were performed to identify factors associated with self-care maintenance, self-care management, and self-care confidence. Variables with *P* < 0.05 in bivariate analyses, together with clinically relevant variables identified from the literature, were entered into the multivariable models. Categorical variables with more than two categories were dummy coded.

Exploratory interaction analyses were conducted to test whether the associations between domain-specific IC impairments (impaired vs. non-impaired) and self-care were moderated by age, NYHA class, and educational level. To mitigate multiplicity, these three moderators were selected based on existing heart-failure self-care frameworks and existing research indicating their clinical relevance. Interaction effects (IC domain  ×  moderator) were tested using PROCESS v4.2 (Model 1) for the continuous moderator (age) and general linear models for the categorical moderators (NYHA class and educational level). Significant interactions were further probed with simple slope analyses and visualized.

Regression diagnostics, model specification, and model fit were evaluated for the linear regression models. To examine potential nonlinearity in the association between composite IC and self-care outcomes, the composite IC score was mean-centered, and a quadratic term was generated from the centered score. Multicollinearity was assessed using tolerance and variance inflation factor (VIF), with VIF < 5 indicating acceptable collinearity. Autocorrelation was assessed using the Durbin-Watson statistic. Linearity and homoscedasticity were evaluated by inspecting scatterplots of standardized residuals against standardized predicted values, and residual normality was assessed using normal P-P plots. Influential observations were examined using studentized residuals (absolute value > 3), Cook's distance (> 1), centered leverage values, and Mahalanobis distance. Model fit was evaluated using R^2^, adjusted R^2^, and the F statistic. All tests were two-sided, and *P* < 0.05 was considered statistically significant.

## 3 Results

### 3.1 Participant characteristics and univariate comparisons of self-care

This study included 215 older adults with heart failure. The mean age was 74.13 years (SD = 7.07), with a range of 60 to 92 years. More than half of the participants were male. Regarding educational level, 42.8% had primary school education or below. Most participants were married (74.9%), and 93.0% had medical insurance coverage. The majority (78.1%) were classified as NYHA class II or III, and 87.4% lived with family or others. Additionally, 69.8% reported a monthly income exceeding RMB 2,000. For comorbidities, 73.5% had hypertension, 65.6% had coronary artery disease, and 73.5% had less than three chronic conditions (Table 1).

**Table 1:** Characteristics of study participants and univariate comparisons of self-care maintenance, management, and confidence across different subgroups (*N* = 215).

Variables	Overall samples	Self-care maintenance		Self-care management		Self-care confidence	
		M ± SD	Statistic	M ± SD	Statistic	M ± SD	Statistic
Age (years)	74.13 ± 7.07						
BMI (Kg/m^2^)	23.91 ± 3.80						
Gender			t = 0.500 *P* = 0.617		t = 0.581 *P* = 0.562		t = 1.548 *P* = 0.123
Male	122 (56.7)	42.84 ± 14.97		35.70 ± 19.03		40.15 ± 20.46	
Female	93 (43.3)	41.79 ± 15.58		34.19 ± 18.46		35.99 ± 18.21	
Education level			F = 6.121 *P* = 0.003		F = 3.876 *P* = 0.022		F = 6.921 *P* = 0.001
Primary school or lower	92 (42.8)	39.16 ± 13.00		32.28 ± 17.31		32.94 ± 17.63	
Junior high school	77 (35.8)	42.55 ± 16.89		34.48 ± 19.78		41.16 ± 20.39	
High school and above	46 (21.4)	48.55 ± 14.72		41.52 ± 18.64		44.48 ± 19.54	
Marital status			t = 2.500 *P* = 0.013		t = 1.663 *P* = 0.098		t = 0.735 *P* = 0.463
Married	161 (74.9)	43.87 ± 15.17		36.27 ± 18.33		38.92 ± 19.12	
Divorced or widowed	54 (25.1)	37.96 ± 14.60		31.39 ± 19.70		36.65 ± 21.01	
Medical expense coverage			t = 0.659 *P* = 0.510		t = 0.152 *P* = 0.879		t = 0.718 *P* = 0.473
Self-funded	15 (7.0)	44.88 ± 15.42		34.33 ± 18.01		34.84 ± 19.20	
Medical insurance	200 (93.0)	42.20 ± 15.22		35.10 ± 18.86		38.61 ± 19.64	
NYHA class			F = 1.104 *P* = 0.333		F = 0.651 *P* = 0.523		F = 0.681 *P* = 0.507
Class II	88 (40.9)	44.20 ± 14.49		35.40 ± 19.41		39.93 ± 20.24	
Class III	80 (37.2)	40.83 ± 14.91		33.38 ± 17.02		36.42 ± 17.91	
Class IV	47 (21.9)	41.63 ± 16.92		37.23 ± 20.43		38.68 ± 21.15	
Living arrangement			t = 1.371 *P* = 0.172		t = 0.891 *P* = 0.374		t = 0.306 *P* = 0.760
Living alone	27 (12.6)	38.64 ± 15.53		32.04 ± 23.50		37.27 ± 23.47	
Living with family/others	188 (87.4)	42.92 ± 15.13		35.48 ± 18.01		38.51 ± 19.03	
Monthly income (CNY)			F = 0.348 *P* = 0.707		F = 1.851 *P* = 0.160		F = 6.914 *P* = 0.001
＜500	37 (17.2)	40.72 ± 15.54		33.38 ± 17.08		30.20 ± 18.83	
500–2,000	28 (13.0)	41.66 ± 15.86		29.46 ± 17.50		32.17 ± 13.98	
＞2,000	150 (69.8)	42.93 ± 15.08		36.50 ± 19.25		41.51 ± 19.90	
Current smoking status			t = 1.089 *P* = 0.277		t = 0.878 *P* = 0.381		t = 0.200 *P* = 0.842
Yes	70 (32.6)	40.76 ± 13.11		33.43 ± 17.60		37.97 ± 19.67	
No	145 (67.4)	43.17 ± 16.11		35.83 ± 19.30		38.54 ± 19.61	
Current drinking status			t = 0.950 *P* = 0.343		t = 1.038 *P* = 0.300		t = 0.739 *P* = 0.461
Yes	12 (5.6)	38.33 ± 14.10		29.58 ± 10.54		34.29 ± 13.38	
No	203 (94.4)	42.62 ± 15.27		35.37 ± 19.10		38.59 ± 19.89	
Number of comorbidities			t = 2.32 *P* = 0.021		t = 0.64 *P* = 0.524		t = −0.26 *P* = 0.799
<3	158 (73.5)	43.81 ± 14.19		35.54 ± 19.03		38.15 ± 18.54	
≥3	57 (26.5)	38.42 ± 17.26		33.68 ± 18.09		38.92 ± 22.39	
Self-care		42.38 ± 15.21		35.05 ± 18.76		38.35 ± 19.59	

Data are presented as mean ± standard deviation or *n* (%).

BMI, body mass index; NYHA, New York Heart Association; CNY, Chinese Yuan.

Comparisons of self-care maintenance, management, and confidence across socio-demographic and clinical characteristics are presented in Table 1. Self-care maintenance was significantly associated with educational level (*P* = 0.003), marital status (*P* = 0.013), and number of comorbidities (*P* = 0.021). Self-care management differed significantly only by educational level (*P* = 0.022). Self-care confidence differed significantly by educational level (*P* = 0.001) and monthly income (*P* = 0.001). No significant differences were observed for gender, medical insurance coverage, NYHA class, living arrangement, smoking, or drinking across any of the three self-care dimensions (all *P* values > 0.05).

### 3.2 Frequency and distribution of IC impairment

The average composite IC score for older adults with heart failure was 3.25 (SD = 1.18). Out of the five areas of IC impairment, 99.5% of participants demonstrated an impairment in at least one IC domain. Impairments were most prevalent in vitality (87.9%, *n* = 189) and cognition (84.2%, *n* = 181). Locomotor and sensory impairments were observed in 72.6% and 49.3% of participants, respectively, whereas psychological impairment was the least common (30.7%). Among all observed domain impairments, 36 individuals (16.7%) experienced losses in all five sub-domains of IC. Additionally, 54 individuals (25.1%) had impairments in 4 domains, 72 individuals (33.5%) had impairments in 3 domains, 34 individuals (15.8%) had impairments in 2 domains, and 18 individuals (8.4%) had impairments in only one domain. Figure 1 depicted the frequency of various combinations of IC impairment aspects with no impairments in the remaining domains. The most frequent occurrence was a simultaneous impairment in the cognition, vitality, and locomotor domains, with a sample size of 40.

**Figure 1:**
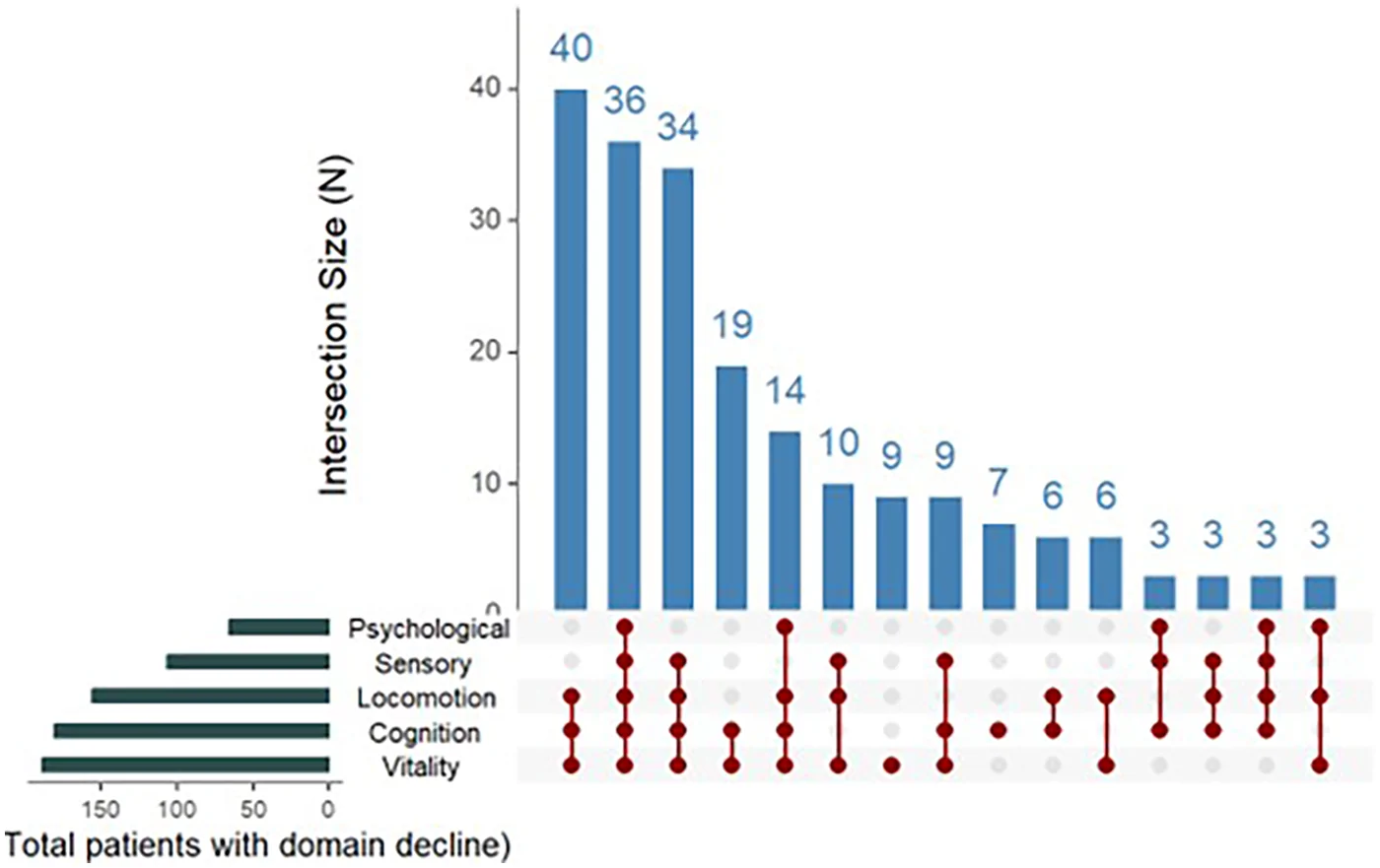
Patterns of concurrent impairments in intrinsic capacity domains among older adults with heart failure. The bars represent the frequency of various combinations of intrinsic capacity domain impairment. The matrix below the bars indicates the specific domains involved in each combination. The connected dots represent the presence of impairment within those domains.

### 3.3 Association of domain-specific IC impairment with self-care

As shown in Table 2, cognitive impairment was significantly associated with reduced self-care maintenance and lower self-care confidence. Locomotor impairment was significantly linked to lower self-care confidence, whereas psychological impairment was associated with both reduced self-care maintenance and lower self-care confidence. Sensory impairment was significantly related to poorer self-care maintenance alone. By contrast, vitality impairment showed no significant association with any of the three self-care dimensions, and none of the five IC domains demonstrated a significant association with self-care management.

**Table 2:** Comparison of self-care with each domain of IC impairment (*N* = 215).

Variables	Self-care maintenance		Self-care management		Self-care confidence	
	M ± SD	Statistic	M ± SD	Statistic	M ± SD	Statistic
Cognition impairment		t = 2.387 *P* = 0.018		t = 1.433 *P* = 0.153		t = 3.626 *P* < 0.001
Not impaired	48.03 ± 16.47		39.26 ± 21.11		49.22 ± 22.70	
Impaired	41.32 ± 14.77		34.25 ± 18.24		36.31 ± 18.30	
Locomotor impairment		t = 1.639 *P* = 0.103		t = 1.779 *P* = 0.077		t = 2.824 *P* = 0.005
Not impaired	45.14 ± 13.35		38.73 ± 17.97		44.39 ± 20.51	
Impaired	41.34 ± 15.77		33.65 ± 18.92		36.07 ± 18.79	
Vitality impairment		t = 0.200 *P* = 0.842		t = 0.292 *P* = 0.771		t = 0.811 *P* = 0.418
Not impaired	42.94 ± 12.66		34.04 ± 19.08		41.27 ± 20.15	
Impaired	42.31 ± 15.56		35.19 ± 18.76		37.95 ± 19.53	
Psychological impairment		t = 3.324 *P* = 0.001		t = 0.142 *P* = 0.887		t = 3.363 *P* < 0.001
Not impaired	44.63 ± 14.33		35.17 ± 18.13		41.27 ± 18.99	
Impaired	37.32 ± 16.01		34.77 ± 20.24		31.76 ± 19.45	
Sensory impairment		t = 3.139 *P* = 0.002		t = 1.902 *P* = 0.059		t = 0.586 *P* = 0.558
Not impaired	45.53 ± 14.58		37.43 ± 18.40		39.12 ± 18.91	
Impaired	39.15 ± 15.24		32.59 ± 18.89		37.56 ± 20.31	

M, mean; SD, standard deviation.

### 3.4 Correlation analysis of socio-demographic variables and composite IC score with self-care

Correlation analysis revealed that age was significantly negatively correlated with self-care maintenance (r = −0.249, 95% CI [−0.370, −0.119], *P* < 0.01), self-care management (r = −0.255, 95% CI [−0.376, −0.125], *P* < 0.01), and self-care confidence (r = −0.184, 95% CI [−0.310, −0.051], *P* < 0.01). The composite IC score was significantly negatively correlated with self-care maintenance (r = −0.272, 95% CI [−0.392, −0.143], *P* < 0.01) and self-care confidence (r = −0.266, 95% CI [−0.386, −0.137], *P* < 0.01), but not with self-care management (r = −0.129, 95% CI [−0.258, 0.005], *P* > 0.05). In contrast, no significant correlations were observed between BMI and self-care maintenance, self-care management, or self-care confidence (all *P* values > 0.05).

### 3.5 Multiple linear regression analysis of factors associated with self-care

Table 3 presented the multiple linear regression models identifying factors associated with self-care maintenance, management, and confidence. Age was significantly and negatively associated with self-care maintenance (B = −0.37, 95% CI [−0.67, −0.07], *P* = 0.014), self-care management (B = −0.64, 95% CI [−1.02, −0.26], *P* = 0.001), and self-care confidence (B = −0.38, 95% CI [−0.77, −0.0004], *P* = 0.049). A higher composite IC score was significantly associated with lower self-care maintenance (B = −2.81, 95% CI [−4.68, −0.95], *P* = 0.003) and lower self-care confidence (B = −3.53, 95% CI [−5.95, −1.11], *P* = 0.004), but not with self-care management (B = −1.08, 95% CI [−3.46, 1.30], *P* = 0.372). Compared with primary school education or below, high school education or above was significantly associated with higher self-care maintenance (B = 9.80, 95% CI [3.87, 15.73], *P* = 0.001), whereas its associations with self-care management (B = 7.39, 95% CI [−0.19, 14.96], *P* = 0.056) and self-care confidence (B = 5.02, 95% CI [−2.69, 12.72], *P* = 0.201) did not reach statistical significance.

**Table 3:** Influencing factors of self-care maintenance, management, and confidence using multiple linear regression analysis (*N* = 215).

Variables	Self-care maintenance			Self-care management			Self-care confidence		
	B[SE] (95% CI)	β	P	B[SE] (95% CI)	β	P	B[SE] (95% CI)	β	P
Constants	78.03 [10.87] (56.60, 99.46)	-	<0.001	82.72 [13.88] (55.35, 110.09)	-	<0.001	70.24 [14.12] (42.39, 98.08)	-	<0.001
Age	−0.37 [0.15] (−0.67, −0.07)	−0.172	0.014	−0.64 [0.19] (−1.02, −0.26)	−0.24	0.001	−0.38 [0.20] (−0.77, −0.0004)	−0.139	0.049
Gender (Female)	0.33 [2.06] (−3.74, 4.39)	0.011	0.874	0.31 [2.63] (−4.88, 5.50)	0.008	0.907	−2.61 [2.68] (−7.89, 2.67)	−0.066	0.330
NYHA class Ⅲ	0.83 [2.36] (−3.82, 5.48)	0.026	0.726	1.38 [3.01] (−4.56, 7.32)	0.036	0.647	0.63 [3.07] (−5.42, 6.67)	0.015	0.838
NYHA class Ⅳ	0.84 [2.82] (−4.71, 6.39)	0.023	0.765	3.47 [3.60] (−3.62, 10.56)	0.077	0.336	3.07 [3.66] (−4.15, 10.28)	0.065	0.403
Junior high school	3.47 [2.40] (−1.26, 8.19)	0.11	0.149	1.17 [3.06] (−4.87, 7.20)	0.030	0.703	4.12 [3.11] (−2.02, 10.26)	0.101	0.187
High school and above	9.80 [3.01] (3.87, 15.73)	0.265	0.001	7.39 [3.84] (−0.19, 14.96)	0.162	0.056	5.02 [3.91] (−2.69, 12.72)	0.105	0.201
Monthly income (500–2,000 CNY)	0.20 [3.67] (−7.03, 7.43)	0.004	0.956	−3.43 [4.68] (−12.66, 5.81)	−0.062	0.465	2.30 [4.76] (−7.10, 11.69)	0.040	0.630
Monthly income (> 2,000 CNY)	−2.95 [2.92] (−8.71, 2.81)	−0.089	0.314	0.35 [3.73] (−7.01, 7.70)	0.008	0.926	7.15 [3.80] (−0.34, 14.63)	0.168	0.061
Comorbidity (≥ 3)	−4.03 [2.32] (−8.59, 0.54)	−0.117	0.084	−0.34 [2.96] (−6.18, 5.49)	−0.008	0.908	1.63 [3.01] (−4.30, 7.57)	0.037	0.588
IC composite score	−2.81 [0.95] (−4.68, −0.95)	−0.219	0.003	−1.08 [1.21] (−3.46, 1.30)	−0.068	0.372	−3.53 [1.23] (−5.95, −1.11)	−0.213	0.004
R^2^	0.169			0.109			0.154		
Adjusted R^2^	0.129			0.065			0.112		
F (P)	4.162 (<0.001)			2.494 (0.008)			3.712 (<0.001)		

B, unstandardized regression coefficient; SE, standard error; β, standardized regression coefficient; 95% CI, 95% confidence interval for B; CNY, Chinese Yuan. Reference categories: Gender (Male), NYHA class (Class II), Education (Primary school or lower), Monthly income (<500 CNY), Comorbidity (<3).

### 3.6 Exploratory interaction analysis of age, NYHA class, and educational level on domain-specific IC impairment and self-care

Exploratory interaction analyses were performed to examine whether the associations between domain-specific IC impairments and self-care differed by age, NYHA class, or educational level. For age, significant interaction effects were found for vitality impairment × age on self-care maintenance (B = −0.986, *P* = 0.041, *Δ*R^2^ = 0.018), locomotor impairment × age on self-care management (B = −1.048, *P* = 0.026, *Δ*R^2^ = 0.022), and psychological impairment × age on self-care management (B = −0.750, *P* = 0.044, *Δ*R^2^ = 0.018) (Supplementary Table S1). No significant interaction effects were observed for age on self-care confidence (Supplementary Table S1). For NYHA class, significant interaction effects were observed for cognitive impairment × NYHA on self-care management (F = 3.154, *P* = 0.045, partial *η*^2^ = 0.029) and locomotor impairment × NYHA on self-care confidence (F = 3.847, *P* = 0.023, partial *η*^2^ = 0.036) (Supplementary Table S2). For education, a significant interaction effect was observed only for sensory impairment × education on self-care management (F = 3.085, *P* = 0.048, partial *η*^2^ = 0.029) (Supplementary Table S3). All other interaction effects were not statistically significant (*P* > 0.05).

To further characterize the significant interaction effects, simple slope analyses were performed (Figure 2). For the vitality impairment × age interaction on self-care maintenance (Figure 2A) and the psychological impairment × age interaction on self-care management (Figure 2B), none of the simple slopes reached statistical significance (all *P* > 0.05), despite significant overall interactions. For the locomotor impairment × age interaction on self-care management (Figure 2C), the negative association was statistically significant in older age (*P* = 0.020), but not in younger (*P* = 0.477) or at the mean age (*P* = 0.115). For the cognitive impairment × NYHA interaction on self-care management (Figure 2D), a significant negative association was observed only in NYHA class II patients (*P* = 0.009). For the locomotor impairment × NYHA interaction on self-care confidence (Figure 2E), significant negative associations were observed in both NYHA class II (*P* = 0.002) and class IV (*P* = 0.020) patients. For the sensory impairment × education interaction on self-care management (Figure 2F), the negative association was significant only in patients with junior high school education (*P* = 0.007).

**Figure 2:**
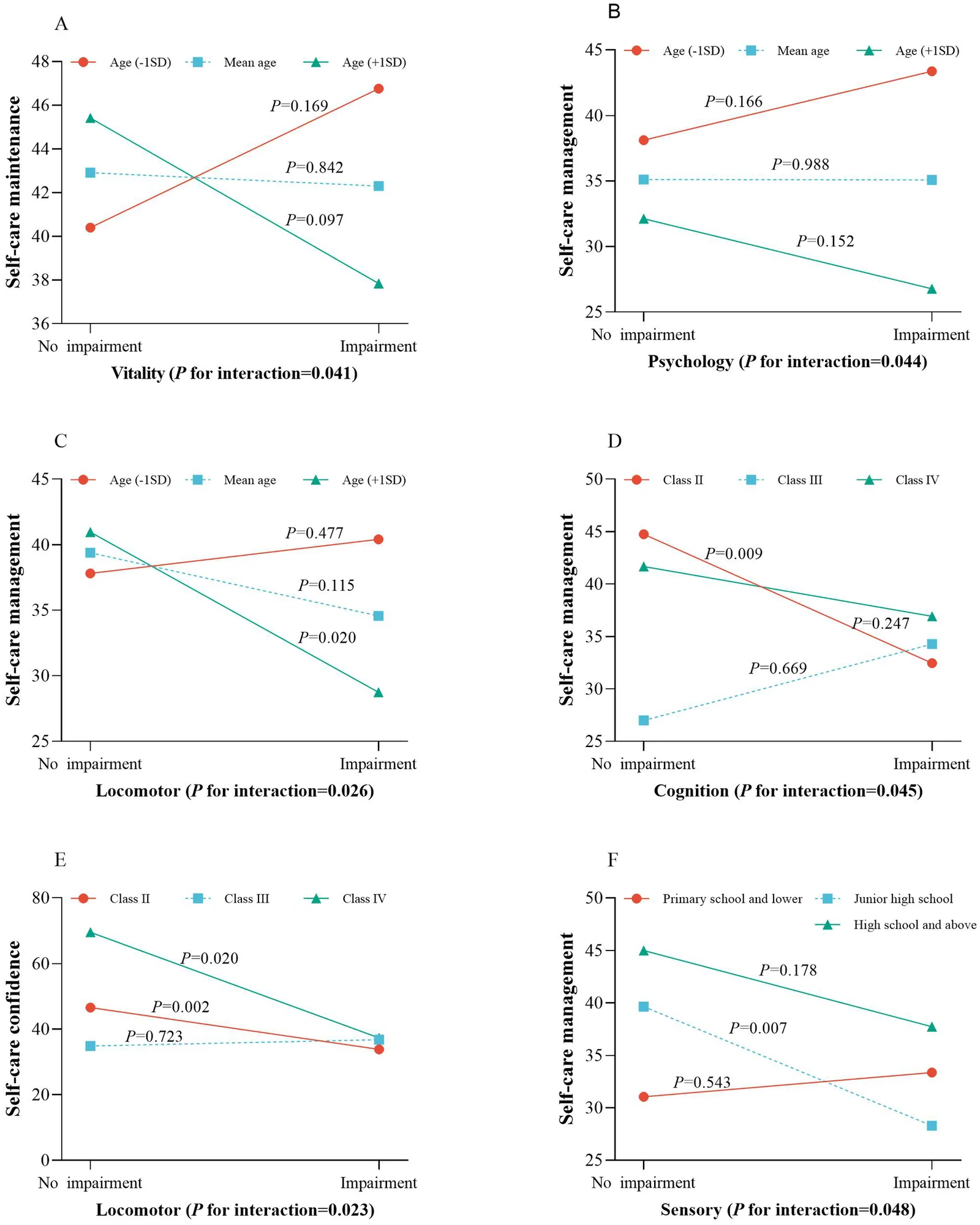
Simple slope analyses of the significant interaction effects between domain-specific IC impairments and age/ NYHA class/ education on self-care. **(A)** Interaction effect of vitality impairment and age on self-care maintenance. **(B)** Interaction effect of locomotor impairment and age on self-care management. **(C)** Interaction effect of psychological impairment and age on self-care management. **(D)** Interaction effect of cognitive impairment and NYHA class on self-care management. **(E)** Interaction effect of locomotor impairment and NYHA class on self-care confidence. **(F)** Interaction effect of sensory impairment and education on self-care management. IC, intrinsic capacity; NYHA, New York Heart Association. The *X*-axis represents the presence or absence of the specific domain-specific IC impairment. The *Y*-axis represents the estimated marginal means of self-care. Lines represent different levels of the moderators. The *P* value for interaction shown below each panel indicates the overall interaction effect. The *P* values displayed on each line indicate the significance of the simple slopes within each subgroup. SD, standard deviation.

### 3.7 Model diagnostics and linearity assessment

Model diagnostics were performed to evaluate the robustness of the three multiple linear regression models (Supplementary Tables S4-S7). All VIF values were below 2.0 (maximum VIF = 1.944), indicating no multicollinearity. Durbin-Watson values ranged from 1.720 to 1.738 (close to 2), suggesting no residual autocorrelation. Although a few studentized residuals exceeded an absolute value of 3 (maximum 3.791), the maximum Cook's distance was 0.075, and centered leverage and Mahalanobis distance values were within acceptable ranges (Supplementary Table S4), indicating that no influential observations were identified. The mean-centered quadratic term for the composite IC score was not significant for self-care maintenance (*P* = 0.442), self-care management (*P* = 0.901), or self-care confidence (*P* = 0.926), supporting a linear relationship between IC and self-care outcomes (Supplementary Tables S5-S7). The normal P-P plot (Supplementary Figures S1-S3) showed that points closely followed the diagonal line, supporting normality of residuals. The scatterplot of standardized residuals versus standardized predicted values (Supplementary Figures S4-S6) displayed no systematic pattern, consistent with homoscedasticity.

## 4 Discussion

Our study found that IC impairment is highly prevalent among older adults with heart failure, with 99.5% of participants exhibiting impairment in at least one IC domain. This finding aligns with previous research reporting a 92.6% prevalence of at least one IC domain impairment in the general elderly population (29). Among the five IC domains, vitality and cognition were most frequently impaired (87.9% and 84.2%, respectively). The high prevalence of vitality impairment aligns with pathophysiology of heart failure, which is closely linked to malnutrition and catabolic states. Neurohormonal dysregulation and systemic inflammatory activation disrupt the balance between anabolic and catabolic processes (30). Heart failure may also exacerbate nutritional deficiencies by altering appetite, food intake, and nutrient malabsorption (31). Similarly, cognitive impairment is a well-documented comorbidity in heart failure, driven by chronic cerebral hypoperfusion, systemic inflammation, neurohumoral activation, microvascular ischemia, and disruption of the blood-brain barrier (32). The most frequent co-occurrence in our sample involved cognition, vitality, and locomotor functions. This contrasts with findings from Liu et al., who identified sensory, vitality, and locomotor functions as the most common pattern in the general older population (14). One possible explanation is the higher burden of shared risk factors for cognitive decline in people with heart failure and the bidirectional relationship between heart failure and cognitive impairment, which may accelerate cognitive deterioration compared with general older adults (33). In addition, vitality in our study was assessed using the MNA-SF, which includes items related to mobility and neuropsychological problems. Thus, the operational definition of vitality partially overlaps with locomotor, cognitive, and psychological domains, which may further contribute to the frequent co-occurrence of cognition-vitality-locomotor impairments in our sample.

A higher composite IC score, reflecting a greater number of impaired domains, was significantly associated with poorer self-care maintenance and lower self-care confidence, but not with self-care management in heart failure. This result differs from findings in older adults, where IC was significantly related to self-management as measured by the Self-Management Ability Scale-30 (14). Differences in study populations and measurement instruments are likely to account for part of this discrepancy. More importantly, the three components of heart failure self-care are conceptually distinct. Self-care maintenance refers to routine behaviors, and therefore relies heavily on the cognitive and physical capacities captured by composite IC. Self-care management, in contrast, is episodic and triggered by symptom occurrence (34). Consequently, its association with a composite IC score can be attenuated or mediated by knowledge and confidence (35). Moreover, a composite IC score may capture broad vulnerability that aligns more closely with everyday functional self-care capacity and perceived ability than with the episodic decision process of management. This distinction may further account for divergent associations of IC impairments with three self-care dimensions.

The associations between IC domains and heart failure self-care were dimension specific. Cognitive impairment was significantly associated with poorer self-care maintenance and lower confidence, but not with management. Deficits in attention, memory, executive function, and processing speed can directly hinder symptom monitoring and complex medication adherence, and undermine perceived self-care ability (36). Psychological impairment showed a similar pattern, being associated with poorer self-care maintenance and confidence. Depressive symptoms are linked to emotional suppression and reduced motivation, which can blur symptom interpretation and reduce treatment adherence (37). Locomotor impairment was associated only with lower self-care confidence. Fatigue, dyspnea, and exercise intolerance commonly seen in locomotor limitations erode physical independence and self-efficacy, without necessarily preventing seated self-care tasks or symptom-response decisions. Sensory impairment was associated only with poorer maintenance, likely because visual and auditory deficits compromise tasks such as reading labels, monitoring diet and fluids, which are essential for routine maintenance.

We observed that older age was significantly associated with poorer self-care maintenance, management, and confidence in older adults with heart failure. This finding aligns with existing evidence indicating worse self-care in elderly populations than their younger counterparts (38). This relationship is supported by the self-management of abilities theory describing that self-management becomes more challenging in later life (39). Given that IC follows a progressive trajectory of deterioration in older adults (40), cardiovascular ageing in heart failure likely synergizes with these age-related biological shifts, accelerating impairment across multiple IC domains (41). Higher educational level was associated with better self-care maintenance in older adults with heart failure, which is supported by findings in heart failure (42). Patients with higher educational levels may better understand and engage with health education, perceive symptoms accurately, and adopt self-care behaviors (42). Moreover, educational level is related to health literacy. Patients with low health literacy may undervalue self-care or struggle to implement proper self-care performance (43). In addition, NYHA class did not show significant main effects on any self-care dimension, which is not consistent with findings that NYHA class III or IV explained 20% of the variance in self-care management (44). This discrepancy may reflect differences in sample characteristics and underscores the need for further investigation of the role of symptom severity in heart failure self-care.

Our exploratory analyses suggested several potential interaction patterns. Age appeared to interact with locomotor impairment in relation to self-care management, with the inverse relationship evident only among the oldest participants. This may indicate that advanced age could amplify the functional impact of mobility limitations, although the effect size was small. Education showed an interaction with sensory impairment, with sensory loss associated with poorer self-care management only among participants with junior high school education. This is consistent with the concept that self-care depends on health literacy and contextual resources that vary by educational level. Exploratory analyses also suggested that NYHA class may modify the associations between IC impairments and self-care. Cognitive impairment may be linked to poorer self-care management in NYHA class II, and locomotor impairment to lower self-care confidence in NYHA classes II and IV. Overall, these findings indicate that age, education, and NYHA class may shape which IC impairments are most relevant to self-care. However, given the multiple interaction tests, borderline P-values, and modest effect sizes, these results should be interpreted cautiously and viewed as hypothesis-generating.

Our findings suggest that IC is a clinically meaningful correlate of self-care in older adults with heart failure, particularly for self-care maintenance and confidence. Routine assessment of IC domains may help identify patients at risk of poorer self-care. Cognitive, psychological, and sensory impairments were associated with poorer maintenance, whereas cognitive, locomotor, and psychological impairments were associated with lower confidence. In contrast, no IC domain was significantly associated with self-care management, suggesting that management may depend on other factors and may require separate intervention strategies. Once IC impairments are identified, coordinated multidisciplinary management may be considered to address multiple domains, and interventions may need to be integrated across domains than delivered in isolation (45). Structured patient education and health counseling may be particularly important for older adults with lower educational attainment or cognitive impairment, who appear at higher risk of poor self-care. Future studies are also needed to clarify the mechanisms linking IC domains to self-care dimensions, and to test whether domain-tailored interventions improve self-care outcomes in older adults with heart failure.

This study offers valuable insights for unravelling the association between IC and self-care among older adults with heart failure. However, several limitations must be considered. First, the use of a convenience sample from two hospitals in Wuxi, China, may affect the generalizability of the findings. Future multi-center studies with larger, more diverse cohorts are needed to validate these associations. Second, the IC and its sub-domains in our study were measured primarily using subjective scales, which may introduce bias. The incorporation of objective biomarkers and performance-based measures in future research would strengthen the assessment. Finally, this was a cross-sectional study. Given that IC is a dynamic ability, longitudinal studies are necessary to further explore its relationship with the factors examined in this study.

## 5 Conclusion

This study reveals a high prevalence of IC impairment among older adults with heart failure, with concurrent impairments in cognition, vitality, and locomotor being the most common domains. A higher composite IC score was associated with poorer self-care maintenance and lower self-care confidence, but not with self-care management. Domain-specific analyses showed that cognitive, psychological, and sensory impairments were associated with poorer self-care maintenance, whereas cognitive, locomotor, and psychological impairments were associated with lower self-care confidence. In addition, older age was significantly associated with poorer self-care maintenance, management, and confidence, and higher educational level was significantly correlated with better self-care maintenance. Therefore, fostering self-care through incorporating routine assessment of IC into the care of heart failure, addressing multimodal impairment, and paying particular attention to the oldest patients and those with lower educational level, might be beneficial. While these findings suggest potential implications for intervention, they could not establish a causal relationship. Future studies should validate these associations in broader populations and evaluate targeted, multidomain interventions to preserve IC and improve self-care in older adults with heart failure.

## Data Availability

The raw data supporting the conclusions of this article will be made available by the authors, without undue reservation.
