# Supplementary material for: Factors associated with self-care in older adults with heart failure presenting with composite and domain-specific intrinsic capacity impairment
Source: Front Cardiovasc Med. 2026 Oct 29;13:1996296. doi: 10.3389/fcvm.2026.1996296 (PMC13754703; doi:10.3389/fcvm.2026.1996296)
Supplement: Supplementary file 1 [file Table1.docx]

**Supplementary materials**

Supplementary Table 1 Exploratory analysis of interaction effects between domain-specific IC impairment and age in self-care maintenance (N = 215)

| **IC impairment × age** | **B** | **SE** | **t** | **P** | **ΔR²** |
| --- | --- | --- | --- | --- | --- |
| Self-care maintenance | | | | | |
| Cognition impairment × age | 0.794 | 0.446 | 1.781 | 0.076 | 0.014 |
| Locomotor impairment × age | -0.307 | 0.385 | -0.797 | 0.426 | 0.003 |
| Vitality impairment × age | -0.986 | 0.480 | -2.055 | 0.041 | 0.018 |
| Psychological impairment × age | -0.217 | 0.296 | -0.732 | 0.465 | 0.002 |
| Sensory impairment × age | 0.354 | 0.289 | 1.223 | 0.223 | 0.006 |
| Self-care management | | | | | |
| Cognition impairment × age | 0.771 | 0.555 | 1.39 | 0.166 | 0.008 |
| Locomotor impairment × age | -1.048 | 0.469 | -2.237 | 0.026 | 0.022 |
| Vitality impairment × age | 0.552 | 0.595 | 0.927 | 0.355 | 0.004 |
| Psychological impairment × age | -0.750 | 0.371 | -2.023 | 0.044 | 0.018 |
| Sensory impairment × age | 0.172 | 0.361 | 0.477 | 0.634 | 0.001 |
| Self-care confidence |  |  |  |  |  |
| Cognition impairment × age | -0.347 | 0.577 | -0.601 | 0.549 | 0.002 |
| Locomotor impairment × age | -0.277 | 0.498 | -0.555 | 0.579 | 0.001 |
| Vitality impairment × age | 0.504 | 0.631 | 0.799 | 0.425 | 0.003 |
| Psychological impairment × age | -0.405 | 0.386 | -1.050 | 0.295 | 0.005 |
| Sensory impairment × age | 0.083 | 0.384 | 0.217 | 0.829 | 0.0002 |

Supplementary Table 2 Exploratory analysis of interaction effects between domain-specific IC impairment and NYHA in self-care maintenance (N = 215)

| **IC impairment × NYHA** | **F** | **P** | **Partial η²** |
| --- | --- | --- | --- |
| Self-care maintenance |  |  |  |
| Cognition impairment × NYHA | 1.032 | 0.358 | 0.010 |
| Locomotor impairment × NYHA | 0.923 | 0.399 | 0.009 |
| Vitality impairment × NYHA | 0.718 | 0.489 | 0.007 |
| Psychological impairment × NYHA | 0.627 | 0.535 | 0.006 |
| Sensory impairment × NYHA | 0.469 | 0.626 | 0.004 |
| Self-care management |  |  |  |
| Cognition impairment × NYHA | 3.154 | 0.045 | 0.029 |
| Locomotor impairment × NYHA | 2.938 | 0.055 | 0.027 |
| Vitality impairment × NYHA | 0.600 | 0.550 | 0.006 |
| Psychological impairment × NYHA | 1.059 | 0.349 | 0.010 |
| Sensory impairment × NYHA | 0.468 | 0.627 | 0.004 |
| Self-care confidence |  |  |  |
| Cognition impairment × NYHA | 0.780 | 0.460 | 0.007 |
| Locomotor impairment × NYHA | 3.847 | 0.023 | 0.036 |
| Vitality impairment × NYHA | 0.079 | 0.925 | 0.001 |
| Psychological impairment × NYHA | 0.67 | 0.513 | 0.006 |
| Sensory impairment × NYHA | 0.61 | 0.544 | 0.006 |

Abbreviations: NYHA, New York Heart Association.

Supplementary Table 3 Exploratory analysis of interaction effects between domain-specific IC impairment and education in self-care maintenance (N = 215)

| **IC impairment × education** | **F** | **P** | **Partial η²** |
| --- | --- | --- | --- |
| Self-care maintenance |  |  |  |
| Cognition impairment × education | 0.734 | 0.481 | 0.007 |
| Locomotor impairment × education | 2.483 | 0.086 | 0.023 |
| Vitality impairment × education | 0.842 | 0.432 | 0.008 |
| Psychological impairment × education | 2.313 | 0.102 | 0.022 |
| Sensory impairment × education | 0.543 | 0.582 | 0.005 |
| Self-care management |  |  |  |
| Cognition impairment × education | 1.663 | 0.192 | 0.016 |
| Locomotor impairment × education | 0.925 | 0.398 | 0.009 |
| Vitality impairment × education | 0.652 | 0.522 | 0.006 |
| Psychological impairment × education | 1.416 | 0.245 | 0.013 |
| Sensory impairment × education | 3.085 | 0.048 | 0.029 |
| Self-care confidence |  |  |  |
| Cognition impairment × education | 0.417 | 0.659 | 0.004 |
| Locomotor impairment × education | 1.925 | 0.148 | 0.018 |
| Vitality impairment × education | 2.110 | 0.124 | 0.020 |
| Psychological impairment × education | 0.955 | 0.387 | 0.009 |
| Sensory impairment × education | 0.715 | 0.490 | 0.007 |

Supplementary Table 4 Residual diagnostic statistics for three multiple linear regression models (N = 215)

| **Diagnostic statistic** | **Self-care maintenance** | **Self-care management** | **Self-care confidence** |
| --- | --- | --- | --- |
| Durbin-Watson | 1.732 | 1.738 | 1.720 |
| Studentized residual, min | -2.801 | -2.486 | -2.088 |
| Studentized residual, max | 3.203 | 3.600 | 3.791 |
| Cook’s distance, max | 0.044 | 0.068 | 0.075 |
| Centered leverage value, max | 0.100 | 0.100 | 0.100 |
| Mahalanobis distance, max | 21.337 | 21.337 | 21.337 |

Notes: Studentized residual with absolute value > 3 indicates a potential outlier. Cook’s distance > 1 indicates an influential observation. Centered leverage value identifies high-leverage points. Mahalanobis distance detects multivariate outliers.
Supplementary Table 5 Multiple linear regression analysis of factors associated with self-care maintenance (N = 215)

| **Variables** | **B (95% CI)** | **β** | **P** | **Tolerance** | **VIF** |
| --- | --- | --- | --- | --- | --- |
| (Constant) | 69.06 (47.32, 90.80) | - | < 0.001 | - | - |
| Age | -0.37 (-0.66, -0.07) | -0.17 | 0.015 | 0.841 | 1.190 |
| Gender (Female) | 0.44 (-3.64, 4.52) | 0.014 | 0.831 | 0.895 | 1.117 |
| NYHA III | 0.60 (-4.10, 5.29) | 0.019 | 0.803 | 0.710 | 1.409 |
| NYHA IV | 0.78 (-4.78, 6.34) | 0.021 | 0.782 | 0.692 | 1.445 |
| Junior high school | 3.67 (-1.09, 8.43) | 0.116 | 0.130 | 0.702 | 1.424 |
| High school and above | 9.89 (3.95, 15.83) | 0.267 | 0.001 | 0.616 | 1.624 |
| Monthly income (500-2000 CNY) | 0.48 (-6.79, 7.76) | 0.011 | 0.896 | 0.610 | 1.640 |
| Monthly income (> 2000 CNY) | -2.70 (-8.50, 3.10) | -0.082 | 0.36 | 0.514 | 1.944 |
| Comorbidity (≥3) | -4.07 (-8.64, 0.51) | -0.118 | 0.081 | 0.897 | 1.115 |
| IC composite score (centered) | -2.92 (-4.81, -1.03) | -0.227 | 0.003 | 0.736 | 1.358 |
| IC composite score² (centered) | -0.47 (-1.69, 0.74) | -0.052 | 0.442 | 0.902 | 1.109 |
| R² | 0.169 |  |  |  |  |
| Adjusted R² | 0.129 |  |  |  |  |
| F | 4.162 (10, 204) |  | < 0.001 |  |  |

Note: B, unstandardized regression coefficient; CI, confidence interval; β, standardized regression coefficient; VIF, variance inflation factor; CNY, Chinese Yuan. Reference categories: Gender (Male), NYHA class (Class II), Education (Primary school or lower), Monthly income (< 500 CNY), Comorbidity (< 3). The IC composite score was mean-centered before computing the quadratic term.

Supplementary Table 6 Multiple linear regression analysis of factors associated with self-care management (N = 215)

| **Variables** | **B (95% CI)** | **β** | **P** | **Tolerance** | **VIF** |
| --- | --- | --- | --- | --- | --- |
| (Constant) | 79.24 (51.43, 107.04) | - | < 0.001 | - | - |
| Age | -0.64 (-1.02, -0.26) | -0.24 | 0.001 | 0.841 | 1.190 |
| Gender (Female) | 0.33 (-4.88, 5.55) | 0.009 | 0.900 | 0.895 | 1.117 |
| NYHA III | 1.33 (-4.67, 7.34) | 0.034 | 0.662 | 0.710 | 1.409 |
| NYHA IV | 3.46 (-3.65, 10.57) | 0.076 | 0.339 | 0.692 | 1.445 |
| Junior high school | 1.21 (-4.88, 7.30) | 0.031 | 0.695 | 0.702 | 1.424 |
| High school and above | 7.40 (-0.19, 15.00) | 0.162 | 0.056 | 0.616 | 1.624 |
| Monthly income (500-2000 CNY) | -3.37 (-12.67, 5.93) | -0.061 | 0.476 | 0.610 | 1.640 |
| Monthly income (> 2000 CNY) | 0.40 (-7.02, 7.82) | 0.010 | 0.916 | 0.514 | 1.944 |
| Comorbidity (≥3) | -0.35 (-6.20, 5.50) | -0.008 | 0.906 | 0.897 | 1.115 |
| IC composite score (centered) | -1.10 (-3.52, 1.31) | -0.07 | 0.369 | 0.736 | 1.358 |
| IC composite score² (centered) | -0.10 (-1.65, 1.45) | -0.009 | 0.901 | 0.902 | 1.109 |
| R² | 0.109 |  |  |  |  |
| Adjusted R² | 0.065 |  |  |  |  |
| F | 2.494 (10, 204) |  | 0.008 |  |  |

Note: B, unstandardized regression coefficient; CI, confidence interval; β, standardized regression coefficient; VIF, variance inflation factor; CNY, Chinese Yuan. Reference categories: Gender (Male), NYHA class (Class II), Education (Primary school or lower), Monthly income (< 500 CNY), Comorbidity (< 3). The IC composite score was mean-centered before computing the quadratic term.

Supplementary Table 7 Multiple linear regression analysis of factors associated with self-care confidence (N = 215)

| **Variables** | **B (95% CI)** | **β** | **P** | **Tolerance** | **VIF** |
| --- | --- | --- | --- | --- | --- |
| (Constant) | 58.73 (30.45, 87.02) | - | < 0.001 | - | - |
| Age | -0.38 (-0.77, 0.00) | -0.139 | 0.050 | 0.841 | 1.19 |
| Gender (Female) | -2.63 (-7.94, 2.68) | -0.067 | 0.329 | 0.895 | 1.117 |
| NYHA III | 0.66 (-5.45, 6.77) | 0.016 | 0.831 | 0.71 | 1.409 |
| NYHA IV | 3.08 (-4.16, 10.31) | 0.065 | 0.402 | 0.692 | 1.445 |
| Junior high school | 4.09 (-2.10, 10.28) | 0.1 | 0.194 | 0.702 | 1.424 |
| High school and above | 5.00 (-2.73, 12.73) | 0.105 | 0.203 | 0.616 | 1.624 |
| Monthly income (500-2000 CNY) | 2.25 (-7.21, 11.72) | 0.039 | 0.640 | 0.610 | 1.640 |
| Monthly income (> 2000 CNY) | 7.11 (-0.44, 14.66) | 0.167 | 0.065 | 0.514 | 1.944 |
| Comorbidity (≥3) | 1.64 (-4.31, 7.59) | 0.037 | 0.587 | 0.897 | 1.115 |
| IC composite score (centered) | -3.51 (-5.97, -1.06) | -0.212 | 0.005 | 0.736 | 1.358 |
| IC composite score² (centered) | 0.08 (-1.50, 1.65) | 0.006 | 0.926 | 0.902 | 1.109 |
| R² | 0.154 |  |  |  |  |
| Adjusted R² | 0.112 |  |  |  |  |
| F | 3.712 (10, 204) |  | < 0.001 |  |  |

Note: B, unstandardized regression coefficient; CI, confidence interval; β, standardized regression coefficient; VIF, variance inflation factor; CNY, Chinese Yuan. Reference categories: Gender (Male), NYHA class (Class II), Education (Primary school or lower), Monthly income (< 500 CNY), Comorbidity (< 3). The IC composite score was mean-centered before computing the quadratic term.

Supplementary Figure 1. Normal P-P plot of standardized residuals for the self-care maintenance regression model.

Supplementary Figure 2. Normal P-P plot of standardized residuals for the self-care management regression model.

Supplementary Figure 3. Normal P-P plot of standardized residuals for the self-care confidence regression model.

Supplementary Figure 4. Scatterplot of standardized residuals against standardized predicted values for the self-care maintenance regression model.

Supplementary Figure 5. Scatterplot of standardized residuals against standardized predicted values for the self-care management regression model.

Supplementary Figure 6. Scatterplot of standardized residuals against standardized predicted values for the self-care confidence regression model.
